# The Effect of Pushing the Support Surface With the Upper Limbs on Angular Changes and Muscle Activity During the Momentum-Transfer Phase of the Sit-to-Stand

**DOI:** 10.7759/cureus.99564

**Published:** 2025-12-18

**Authors:** Naoto Inoue, Tomohito Ijiri, Toshiaki Suzuki

**Affiliations:** 1 Graduate School of Health Sciences, Graduate School of Kansai University of Health Sciences, Osaka, JPN; 2 Department of Rehabilitation, Kiba Hospital, Medical Corporation, Juzankai, Osaka, JPN

**Keywords:** angular change, momentum-transfer phase, muscle activity, sit-to-stand, upper limb support

## Abstract

Introduction

The sit-to-stand (STS) transition is a movement in which the body’s center of gravity shifts upward from a seated to a standing position while maintaining balance. Previous studies have reported that this movement is significantly affected by the presence or absence of upper limb support. The momentum transfer phase (Phase II) of STS begins at the moment of buttocks lift off and continues until the ankle joint reaches maximum dorsiflexion. This phase is considered critical, as it requires maximal muscle activity to maintain stability during elevation of the center of gravity while kinetic energy is transferred from the trunk to the lower limbs. Therefore, upper limb support during STS may play an especially important role in Phase II. Based on this, the objective of the present study was to clarify the effect of the upper limb pushing-off action on Phase II of the STS movement.

Methods

Ten healthy adult males (mean age: 25.9 ± 2.7 years; height: 175.5 ± 4.4 cm; weight: 69.2 ± 5.8 kg) with no orthopedic or neurological problems participated. Tests were conducted to examine differences between two conditions: with upper-limb support pushing against the support surface and without upper-limb support, in terms of joint angle changes and root mean square (RMS) of muscle activity during Phase II of the STS. Normality was assessed using the Shapiro-Wilk test; as normality was rejected, comparisons were performed using the Wilcoxon signed-rank test, and effect sizes were calculated. Additionally, correlations between upper-limb pressure and angular changes and RMS during Phase II with upper-limb support were examined.

Results

In Phase II of the STS, when comparing the presence and absence of upper limb support, the RMS of the vastus lateralis was significantly smaller with support.

Conclusions

Pressing the support surface with the upper limbs during Phase II of the STS reduced vastus lateralis muscle activity compared to the condition without upper-limb support, while no significant changes were observed in angular movement. Upper-limb pressure increased during Phase II and was directed externally, posteriorly, and downward. These findings indicate a relationship between angular changes, muscle activity, and upper-limb support during Phase II of the STS.

## Introduction

The sit-to-stand (STS) is an action that involves shifting the body’s center of gravity upward from a seated to a standing position without losing balance [1], and it is considered the most frequently performed movement in daily life [2]. STS can be divided into three phases [3]: the flexion phase (Phase I) begins at the start of the motion and continues until just before the buttocks lift off the chair seat; the momentum-transfer phase (Phase II) begins simultaneously with buttocks lift off and continues until the ankle joint reaches maximum dorsiflexion; and the extension phase (Phase III) begins immediately after maximum ankle dorsiflexion and continues until hip extension, knee extension, and trunk extension are completed.

Individuals who have difficulty performing STS are at a higher risk of falling during walking and may require assistance with daily activities [4]. Furthermore, the inability to perform STS has been associated with increased mortality in the elderly [5]. These findings indicate that STS is a mechanically complex and challenging movement that is essential for performing activities of daily living.

When lifting the buttocks is difficult, it is sometimes possible to achieve buttocks lift off by pushing against the bed or support surface with the upper limbs without adjusting seat height or foot position. This raises the question of what effect pushing the support surface with the upper limbs has on STS performance.

Kinetic analyses of STS have been conducted using technologies such as force plates, video analysis, optoelectronic systems, goniometry, and accelerometers. It has been reported that STS performance is strongly influenced by chair seat height, the presence or absence of upper-limb support, and foot position [5], suggesting that various conditions affect how STS is performed.

In this study, we focused on the role of upper-limb support during STS. Upper-limb support can be provided through armrests, support surfaces, or the area above the knees. Previous studies have reported that using armrests during STS reduces the hip extension moment required for the movement by approximately 50% [6]. While some studies have examined the presence or absence of armrests during STS [7], no previous studies have analyzed pressure changes associated with actively pushing the support surface with the upper limbs.

Phase II of the STS has been identified as a critical stage requiring maximum muscle activity to maintain stability during elevation of the center of gravity, accompanying the transfer of kinetic energy from the trunk to the lower limbs [8]. This suggests that upper-limb support may play a particularly important role during Phase II. Therefore, the objective of this study was to clarify the effects of pushing the support surface with the upper limbs on Phase II of the STS. Understanding these factors may help identify contributors to difficulty in buttocks lift off when upper-limb support is challenging, thereby informing appropriate interventions for functional impairments.

## Materials and methods

Subjects

The subjects were 10 healthy adults with no orthopedic or neurological problems. Previous reports indicate that a hip flexion range of motion of 110° [9,10] and an ankle dorsiflexion range of motion of 20° [11,12] are required to perform the STS. Based on this, participants with hip flexion less than 110° or ankle dorsiflexion less than 20° were excluded. This study was conducted in accordance with the ethical standards of the Declaration of Helsinki and was approved by the Kansai University of Health Sciences Research Ethics Review Committee (approval number 24-22). All participants received written explanations regarding the study’s purpose, procedures, and handling of personal information and provided informed consent after confirming their full understanding.

Methods

In this study, participants performed STS under two conditions. In the first condition, they pushed against a support surface with their upper limbs, and joint motion and muscle activity were measured during Phase II of the STS. Participants were instructed to “push off the support surface with your upper limbs to stand up.” In the second condition, participants stood up without pressing down on the support surface with their upper limbs. They were instructed to “place your upper limbs on the support surface without pressing down, and stand up.”

Markers were attached to the body surface, and participants performed STS from a seated position. Three UM-CAT II three-dimensional motion analysis devices (Unimec Co., Ltd., Tokyo, Japan) and the KineAnalyzer three-dimensional motion analysis system (Kissei Comtec Co., Ltd., Nagano, Japan) were used to analyze angular changes of the trunk, pelvis, hip, knee, ankle, shoulder, and elbow joints. Cameras were positioned to capture participants from head to toe, and the distance between participants and the cameras was maintained consistently.

Angle measurement

**Figure 1:**
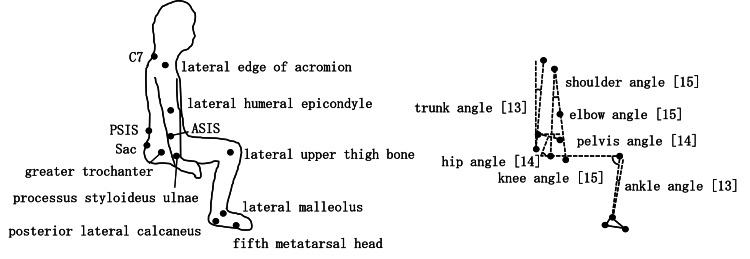
Marker attachment positions and angle measurements Angles were measured based on previously published methods [13-15], and the corresponding reference numbers for each measurement are indicated within the figure. Participants performed the STS movement with the markers attached as shown. ASIS, anterior superior iliac spine; PSIS, posterior superior iliac spine; STS, sit-to-stand

Markers were attached to the following anatomical landmarks: lateral end of the acromion, lateral epicondyle of the humerus, olecranon process of the ulna, spinous process of the seventh cervical vertebra, midpoint of the sacral crest, anterior superior iliac spine (ASIS), posterior superior iliac spine (PSIS), greater trochanter, lateral epicondyle of the femur, lateral malleolus, posterior lateral aspect of the calcaneus, and head of the fifth metatarsal bone [13-15]. In a preliminary study, we confirmed that the double-sided tape used for marker attachment did not peel off the skin during STS. Using these markers, we calculated the trunk, pelvic, hip, knee, ankle, shoulder, and elbow joint angles (Figure 1) [13-15].

The trunk angle was calculated as the angle between the vertical axis and the line connecting the midpoint of C7 and the sacral crest. The pelvic angle was defined as the angle between the horizontal plane and the line connecting the ASIS and the PSIS. The hip angle was calculated as the angle formed between a line perpendicular to the ASIS-PSIS line and the line connecting the greater trochanter and the lateral femoral condyle. The knee joint angle was defined as the angle formed by the line connecting the greater trochanter, lateral femoral condyle, and lateral malleolus. The ankle joint angle was calculated as the angle formed between a line perpendicular to the line connecting the posterior lateral calcaneus and the head of the fifth metatarsal bone, and the line connecting the lateral malleolus and the lateral femoral condyle. The shoulder joint angle was defined as the angle formed by the line connecting the lateral epicondyle of the humerus, the lateral end of the acromion, and the greater trochanter. The elbow joint angle was calculated as the angle formed by the line connecting the lateral end of the acromion, the lateral epicondyle of the humerus, and the olecranon process of the ulna.

Muscle activity measurement

Three-dimensional motion analysis equipment and electromyography (EMG) were synchronized, and measurements were taken simultaneously. EMG data were recorded using a telemetry-type surface electromyograph, MQ-8 (Kissei Comtec Co., Ltd.), with Vital Recorder2 software (Kissei Comtec Co., Ltd.) at a sampling frequency of 1 kHz. The recorded data were analyzed using EMG analysis software BIMUTAS-Video (Kissei Comtec Co., Ltd.).

Before EMG measurement, the skin at the electrode attachment sites was thoroughly cleaned using Skin Pure (Nihon Kohden Corporation, Tokyo, Japan). Disposable LecTrode electrodes (Ag/Ag-Cl, Advance Corporation) with a diameter of 8 mm and an inter-electrode spacing of 15 mm were attached, and muscle activity was recorded using the bipolar method. EMG electrodes were placed on eight sites: multifidus, longissimus, upper fibers of the gluteus maximus, lower fibers of the gluteus maximus, rectus femoris, vastus lateralis, soleus, and the lateral head of the triceps brachii [16,17].

Electrode placement was performed as follows. The multifidus muscle electrode was placed on the muscle belly along the line connecting the PSIS, between the spinous processes of L1 and L2 at the level of L5. The longissimus electrode was placed on the muscle belly 2 transverse fingerbreadths lateral to L1. The upper fibers of the gluteus maximus were recorded from the muscle belly 2 transverse fingerbreadths above the midpoint of the line connecting the PSIS and the inferior end of the greater trochanter, while the lower fibers were placed directly below the midpoint of the same line. The rectus femoris electrode was placed on the muscle belly at the midpoint of the line connecting the ASIS and the superior border of the patella. The vastus lateralis electrode was placed on the muscle belly at the two-thirds point along the line connecting the anterior border of the ilium and the lateral border of the patella. The soleus electrode was positioned on the muscle belly at the two-thirds point along the line connecting the medial epicondyle of the femur and the medial malleolus. The triceps brachii electrode was placed on the muscle belly at the midpoint between the posterior angle of the acromion and the olecranon, two finger-widths lateral to the midpoint.

Measurement regulations

The starting position was aligned by adjusting the chair height to match the length of the lower leg. Joint angles at the start were set to 90° hip flexion and 90° knee flexion [18], foot width was set to match shoulder width [19], and the line of sight was directed toward a target at eye level [20]. Upper limb support was implemented under two conditions. In Condition 1, participants were instructed to push the support surface with their upper limbs during STS while keeping their hands on the support surface. In Condition 2, participants were instructed to stand up without pressing on the support surface, with their hands merely resting on it.

**Figure 2:**
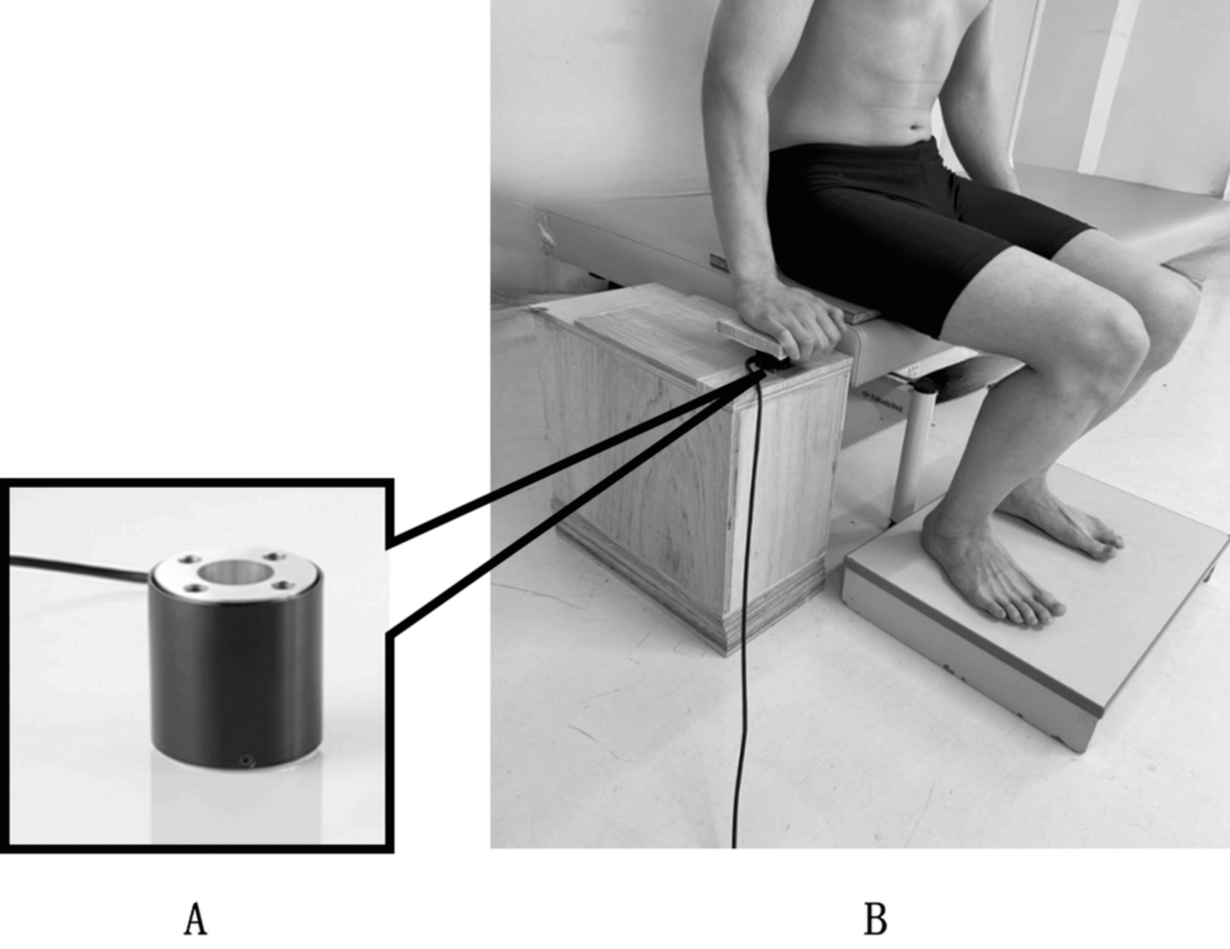
Fixation of the small three-component force transducer and upper limb support position (A) Small three-component force transducer. (B) Method for securing the transducer in place.

A small three-component force transducer TL3B05 (Tech Gihan Co., Ltd., Kyoto, Japan) was used to measure forces in the left-right, front-back, and up-down directions. The sensor was mounted on an aluminum plate and secured in place, with its position aligned to the bed height and the sensor center positioned at the center of the participant’s left hand (Figure 2).

The time required to complete the STS movement was set to two seconds using a metronome [21], and this timing was output as a BNC signal via the audio signal output device EMC-S-BNC (East Medic Co., Ltd., Ishikawa, Japan). Participants were instructed to maintain the shoulder joint in slight abduction and the elbow joint in slight flexion when pushing against the support surface. Hand placement on the support surface was aligned with the proximal interphalangeal joints of the index, middle, and ring fingers (Figure 2). Sitting depth was adjusted to achieve the target elbow angle and hand position.

**Figure 3:**
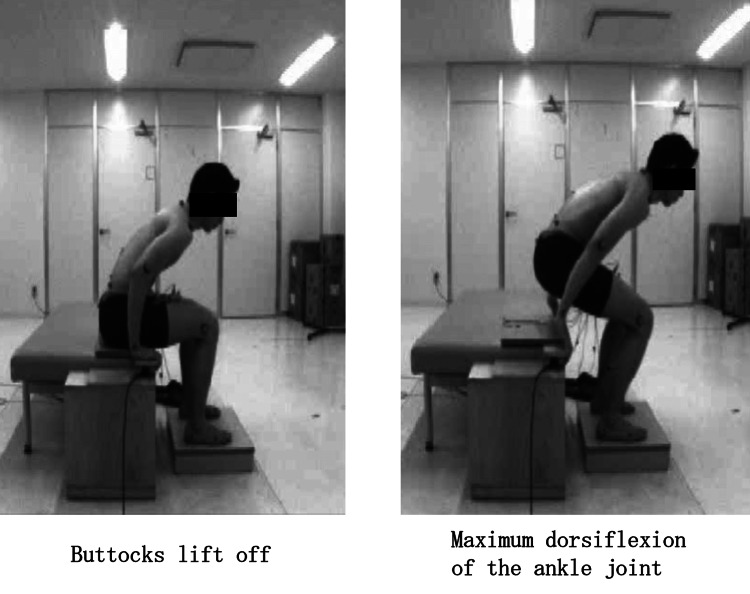
Phase II Phase II was defined as the time from buttocks lift off to the point of maximum dorsiflexion of the ankle joint.

Each condition was measured three times (totaling six measurements), and the average of the three trials per condition was calculated. A one-minute rest period was provided between conditions, and the order of measurements was randomized. A foot switch installed on the seat surface was synchronized with the telemetry-type surface electromyograph MQ-8 to confirm the timing of buttocks lift off on the EMG. The foot switch was positioned under the ischial tuberosity of each participant. A board was placed on the seat surface to harden it, ensuring appropriate foot switch response during Phase II. The timing of Phase II and the maximum dorsiflexion of the ankle joint were entered into the EMG data to synchronize each phase (Figure 3).

Note that Phase II begins at buttocks lift off and ends at the point of maximum dorsiflexion of the ankle joint.

Data analysis

To analyze how pushing the support surface with the upper limbs affects angular changes during Phase II of the STS, angular changes in the trunk, pelvis, hip joint, knee joint, ankle joint, shoulder joint, and elbow joint were calculated from the three-dimensional motion analysis data.

For EMG, because the duration of Phase II varied among subjects, the root mean square (RMS) was used for analysis to accommodate differing time intervals. RMS values were calculated for the multifidus, longissimus, upper fibers of the gluteus maximus, lower fibers of the gluteus maximus, rectus femoris, vastus lateralis, soleus, and lateral head of the triceps brachii during Phase II of the STS.

For the three-component force data, pressures in the left-right, front-back, and up-down directions were calculated at the time of buttocks lift off and at the point of maximum ankle dorsiflexion, with units expressed in kilograms.

Statistical analysis

Statistical analyses were performed using modified R Commander (Version 4.4.1; Graduate School of Health Sciences, Hirosaki University, Aomori, Japan). Differences between the two groups, one with upper limb support pushing against the support surface and the other without upper limb support, were assessed in terms of angular changes and RMS during Phase II of the STS. Normality was tested using the Shapiro-Wilk test; because normality was rejected, the Wilcoxon signed-rank test was applied, and effect sizes were calculated. Additionally, correlations between upper limb pressure and both angular changes and RMS during Phase II with upper limb support were examined. The significance level was set at 5%.

Hypothesis

We hypothesized that, compared to the condition without upper limb support, upper limb support would increase the magnitude of trunk forward tilt, pelvic anterior tilt, hip flexion, and ankle dorsiflexion angles during Phase II of the STS. Furthermore, we hypothesized that muscle activity in the multifidus, longissimus, upper gluteus maximus, lower gluteus maximus, soleus, and lateral head of the triceps brachii would increase with upper limb support, whereas activity in the rectus femoris and vastus lateralis would decrease during Phase II.

## Results

Participants

All participants were male, with a mean age of 25.9 ± 2.7 years, a mean height of 175.5 ± 4.4 cm, and a mean weight of 69.2 ± 5.8 kg.

Angular change

**Figure 4:**
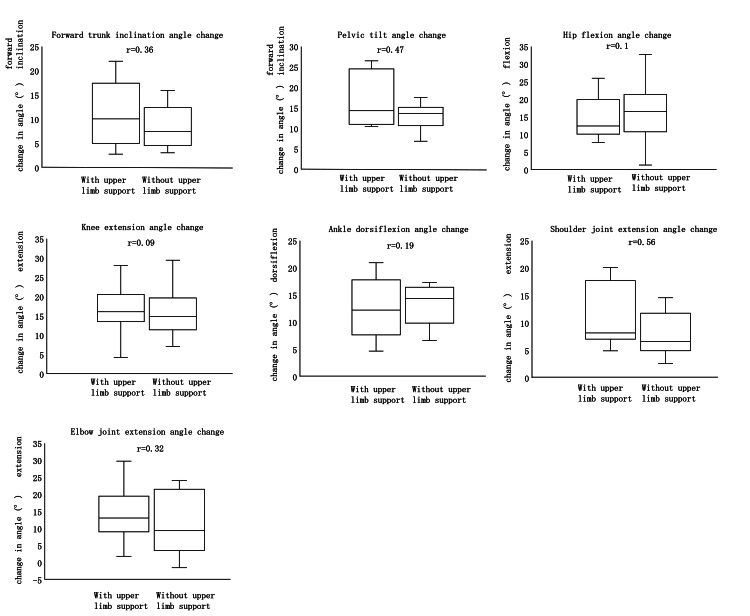
Angular change in Phase II

During Phase II of the STS, no significant differences were observed in trunk angle, pelvic angle, hip joint angle, knee joint angle, ankle joint angle, shoulder joint angle, or elbow joint angle between the upper limb support and no upper limb support conditions (Figure 4).

Muscle activity

**Figure 5:**
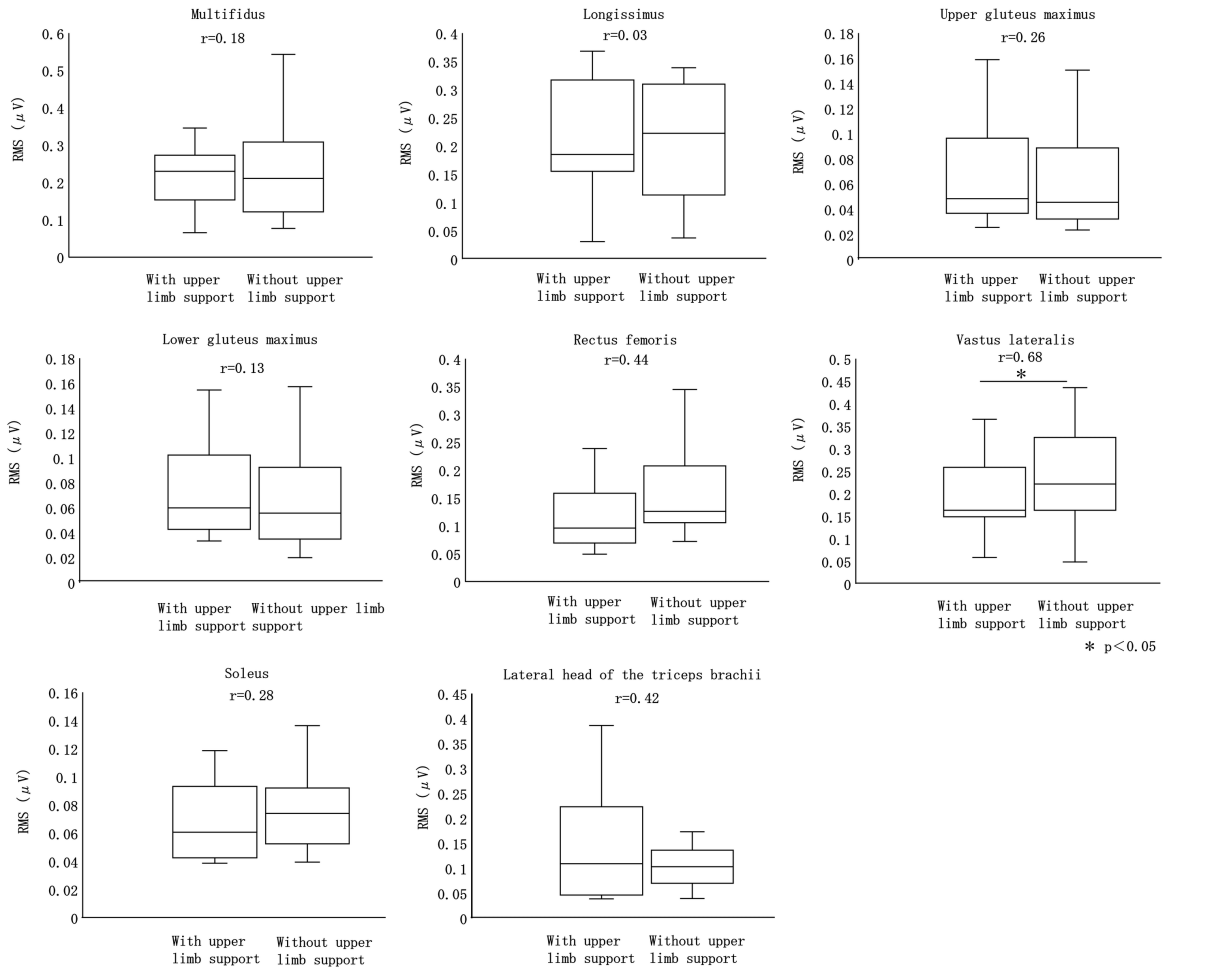
RMS in Phase II RMS, root mean square

During STS Phase II, the RMS of the vastus lateralis was significantly lower with upper limb support compared to without support. No significant differences were observed in the multifidus, longissimus, upper fibers of the gluteus maximus, lower fibers of the gluteus maximus, rectus femoris, soleus, or lateral head of the triceps brachii (Figure 5).

Upper limb pressure

**Figure 6:**
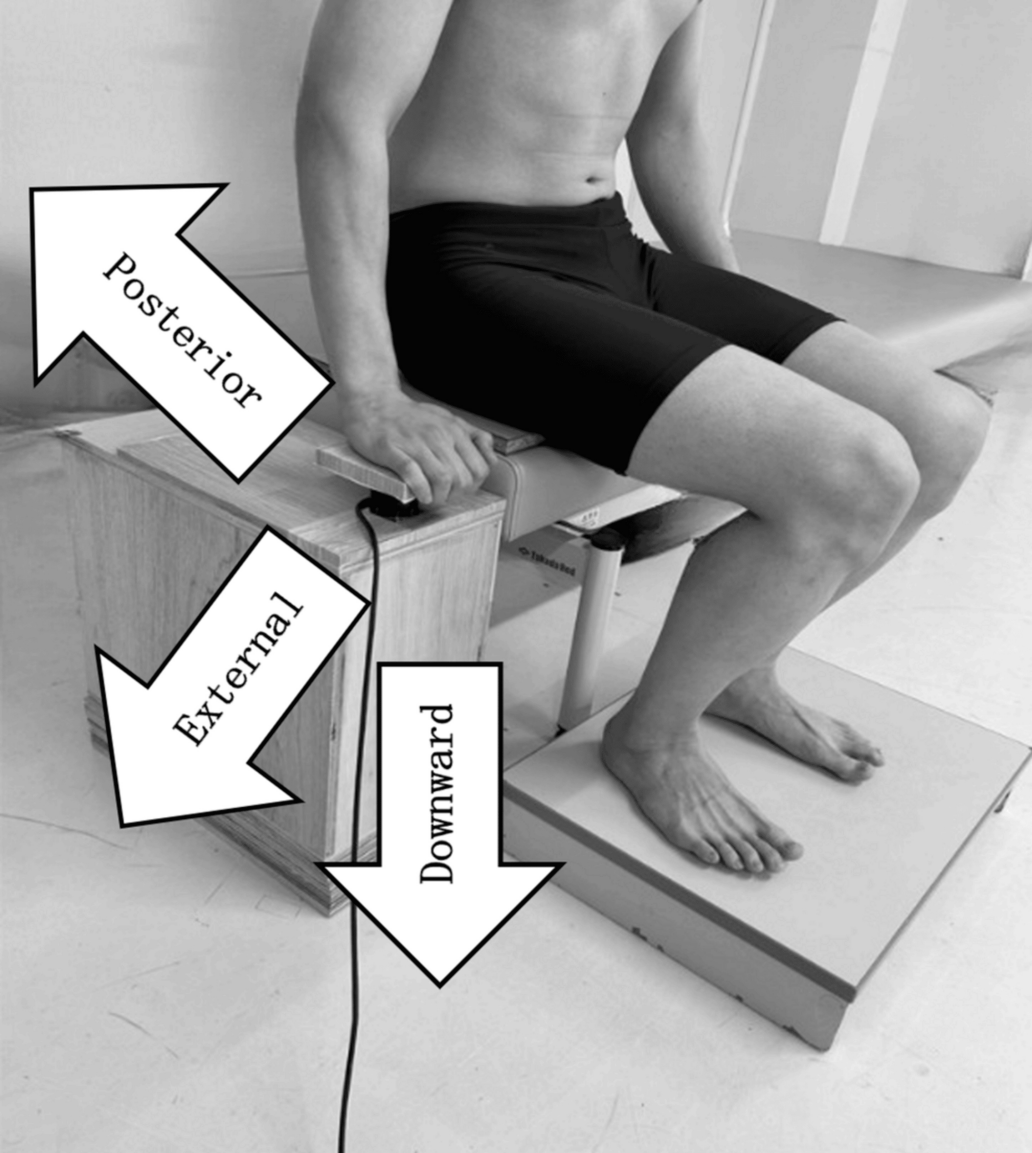
Direction of upper limb pressure During Phase II of the STS, pressure was exerted externally, posteriorly, and downward.

**Figure 7:**
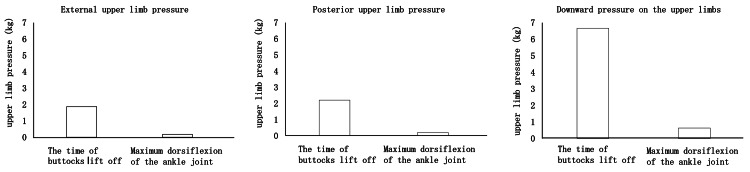
Upper limb pressure during Phase II

During Phase II of the STS, upper limb pressure was directed externally, posteriorly, and downward. This pattern of pressure was maintained from the time of buttocks lift off to the point of maximum ankle dorsiflexion. Moreover, across all three directions of upper limb pressure, the downward component consistently exhibited the greatest magnitude, both at buttocks lift off and at maximum ankle dorsiflexion (Figure 6, Figure 7).

Correlation between upper limb pressure, angular changes, and muscle activity at the time of buttocks lift off

**Figure 8:**
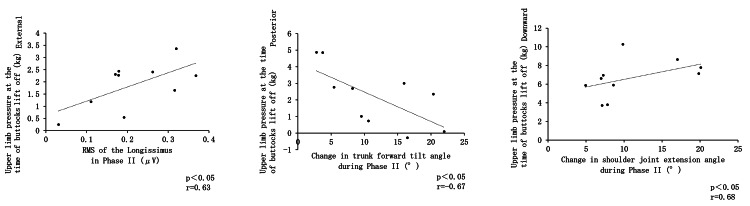
Correlation between upper limb pressure, angular changes, and muscle activity at the time of buttocks lift off

A significant positive correlation was observed between external upper limb pressure at the time of hip buttocks lift off and the RMS of the longissimus muscle during Phase II. Additionally, a significant negative correlation was found between posterior upper limb pressure at buttocks lift off and the change in trunk forward tilt angle during Phase II. Furthermore, a significant positive correlation was observed between downward upper limb pressure at buttocks lift off and the change in shoulder joint extension angle during Phase II (Figure 8).

Correlation between upper limb pressure, angular changes, and muscle activity at the time of maximum ankle dorsiflexion

**Figure 9:**
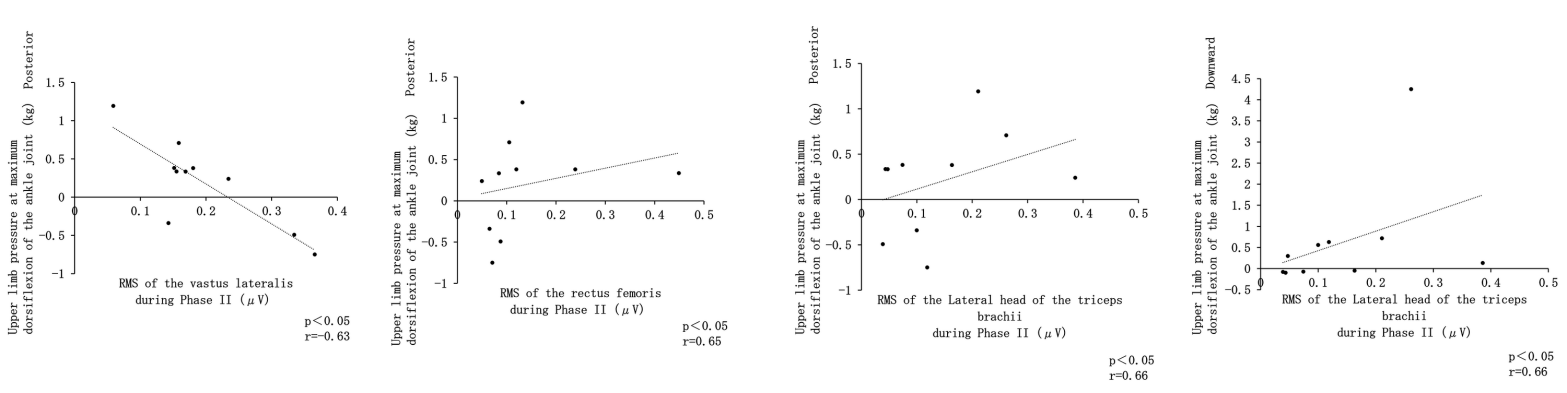
Correlation between upper limb pressure, angular changes, and muscle activity at the time of maximum ankle dorsiflexion

A significant negative correlation was observed between posterior upper limb pressure at the point of maximum ankle dorsiflexion and the RMS of the vastus lateralis during Phase II. In contrast, posterior upper limb pressure at this point showed a significant positive correlation with the RMS of the rectus femoris. Additionally, posterior upper limb pressure at maximum ankle dorsiflexion was positively correlated with the RMS of the lateral head of the triceps brachii during Phase II. Furthermore, downward upper limb pressure at maximum ankle dorsiflexion also exhibited a significant positive correlation with the RMS of the lateral head of the triceps brachii (Figure 9).

## Discussion

In summary, during Phase II of the STS, pressing the support surface with the upper limbs resulted in reduced muscle activity in the vastus lateralis compared to the condition without upper limb support, while no differences were observed in angular changes. Upper limb pressure during Phase II was directed externally, posteriorly, and downward. Based on these findings, the relationships among angular changes, muscle activity, and upper limb pressure during Phase II of the STS were further examined.

Relationship between angular changes, muscle activity, and upper limb pressure at the point of buttocks lift off

Upper Limb Pressure Toward the Outside at the Point of Buttocks Lift Off and RMS of the Longest Muscle During Phase II

Previous studies have suggested that using upper limb support during STS reduces trunk forward tilt and decreases muscle activity in the longissimus muscle [22,23]. However, in this study, a significant positive correlation was observed between external upper limb pressure at buttocks lift off and the RMS of the longissimus muscle during Phase II. The role of the erector spinae muscles in STS includes early contraction during the flexion phase and trunk extension during the extension phase, thereby helping to inhibit excessive trunk forward tilt [14]. The erector spinae muscles comprise the erector spinae, iliocostalis, and spinalis muscles. The erector spinae runs extensively along the spine and acts as the primary muscle generating trunk extension torque [24], while the iliocostalis and spinalis muscles function as stabilizing elements that support the transition from trunk forward tilt to standing during STS [25]. From this, the erector spinae is considered to play the largest role in inhibiting trunk forward tilt. Additionally, comparisons of STS with and without assistive devices such as exoskeletons have shown that although joint angles remain similar, muscle activity in the erector spinae is increased when assistive devices are not used [26]. Therefore, generating external upper limb pressure with both arms at the point of buttocks lift off may inhibit trunk forward tilt by increasing erector spinae muscle activity without altering joint angles.

Posterior Upper Limb Pressure at the Point of Buttocks Lift Off and Changes in Trunk Forward Tilt Angle During Phase II

A study investigating the effects of handrail height on joint motion, center of gravity displacement, and ground reaction forces during STS in 16 healthy young adults and 25 elderly individuals requiring long-term care reported that pulling on a high handrail during STS reduces forward trunk tilt by generating forward and upward reaction forces from the handrail [23]. This finding suggests a relationship between upper limb pressure and trunk forward tilt during STS. In the present study, a negative correlation was observed between posterior upper limb pressure at buttocks lift off and changes in trunk forward tilt angle during Phase II. When trunk forward tilt is minimal, the reduced forward propulsive force may necessitate increased posterior upper limb pressure to assist in generating forward propulsion.

Upper Limb Pressure Downward at the Time of Buttocks Lift Off and the Change in Shoulder Joint Extension Angle During Phase II

At the point of hip buttocks lift off, a significant positive correlation was observed between downward upper limb pressure and the change in shoulder joint extension angle during Phase II. At this moment, shoulder joint extension and elbow joint flexion position the forearm nearly perpendicular to the support surface, which is likely to increase downward upper limb pressure.

Angular changes, muscle activity, and upper limb pressure at the point of maximum ankle dorsiflexion

Upper Limb Pressure Toward the Posterior at the Point of Maximum Ankle Dorsiflexion and RMS of the Lateral Vastus Muscle During Phase II

A significant negative correlation was observed between posterior upper limb pressure at the point of maximum ankle dorsiflexion and the RMS of the vastus lateralis during Phase II. At this point, the anterior tilt of the lower leg increases due to ankle dorsiflexion, causing the body to move downward. Since the maximum dorsiflexion point of the ankle marks the end of Phase II, knee extension is required to achieve standing thereafter. Posterior upper limb pressure assists knee joint extension, resulting in decreased RMS of the vastus lateralis, as this direction of pressure aligns with the rotational axis of knee joint extension.

Posterior Upper Limb Pressure at the Point of Maximum Ankle Dorsiflexion and the RMS of the Rectus Femoris During Phase II

When posterior upper limb pressure increased during Phase II, a smaller change in trunk forward tilt was observed at the start of the phase. This suggests that, at the point of maximum ankle dorsiflexion, the rectus femoris increased its muscle activity to facilitate trunk forward tilt through hip flexion.

Upper Limb Pressure Toward the Posterior at the Point of Maximum Ankle Dorsiflexion and RMS of the Lateral Head of the Triceps Brachii During Phase II

Muscle activity of the triceps brachii, which functions in elbow extension, is considered to have increased to generate posterior upper limb pressure at the point of maximum ankle dorsiflexion.

Upper Limb Pressure Toward the Bottom at the Point of Maximum Ankle Dorsiflexion and RMS of the Lateral Head of the Triceps Brachii During Phase II

It is hypothesized that triceps brachii activity increased to generate downward upper limb pressure at the point of maximum ankle dorsiflexion, facilitating the push against the support surface.

Limitations of the study

This study analyzed only Phase II and did not examine its relationship with the flexion phase. Additionally, the subjects were limited to young healthy males, leaving it unclear whether results would differ between sexes or across age groups. The small sample size also limits the generalizability of the findings to clinical populations.

## Conclusions

In this study, Phase II of the STS was compared in 10 healthy adult males with and without upper limb support. With upper limb support, no significant differences were observed in angular changes during Phase II compared to the condition without upper limb support; however, muscle activity of the vastus lateralis was reduced. At the point of buttocks lift off, external upper limb pressure was associated with the RMS of the erector spinae, posterior upper limb pressure was associated with changes in trunk forward tilt, and downward upper limb pressure was associated with changes in shoulder joint extension. At the point of maximum ankle dorsiflexion, posterior upper limb pressure was associated with RMS of the rectus femoris and vastus lateralis, while both posterior and downward upper limb pressures were associated with RMS of the lateral head of the triceps brachii.
